# Predictors of Methotrexate Success and Fertility Outcomes in Tubal Ectopic Pregnancy: A Retrospective Cohort Study

**DOI:** 10.3390/medicina61061058

**Published:** 2025-06-09

**Authors:** Elisa Scarpelli, Vito Andrea Capozzi, Licia Roberto, Asya Gallinelli, Alessandra Pezzani, Michela Monica, Roberto Berretta

**Affiliations:** Department of Obstetrics and Gynecology, University Hospital of Parma, 43125 Parma, Italy

**Keywords:** ectopic pregnancy, methotrexate, salpingectomy, tubal pregnancy, tubal rupture, maternal death, gestational sac diameter, beta-HCG

## Abstract

*Background and Objectives*: Ectopic pregnancy (EP) is a potentially life-threatening condition and the leading cause of maternal mortality in the first trimester. Although both surgical and medical approaches are effective, selection criteria for Methotrexate (MTX) treatment remain inconsistent across international guidelines. Additionally, limited data on long-term reproductive outcomes are available. *Materials and Methods*: We conducted a single-center retrospective cohort study of 107 patients diagnosed with tubal EP and treated at the Obstetrics and Gynecology Unit of the University Hospital of Parma between 2019 and 2023. MTX (50 mg/m^2^) was offered to patients with β-hCG < 5000 mIU/mL, sac diameter < 40 mm, and no embryonic cardiac activity or hemoperitoneum; others underwent salpingectomy. Treatment outcomes, predictors of MTX success, and fertility outcomes were analyzed. *Results*: Medical treatment was offered to 36 patients (33.6%), with an overall success rate of 72%: in total, 20 resolved after a single dose and 6 after a second dose. Surgical conversion was necessary in 10 patients. The remaining 71 patients (66.4%) underwent primary salpingectomy. Initial β-hCG levels and gestational age did not significantly predict MTX failure (*p* 0.14 and 0.73, respectively), whereas gestational sac diameter was identified as a reliable predictor of treatment success (*p* = 0.01). In particular, a gestational sac maximum diameter of <2 cm emerged as a positive factor for MTX success (OR 1.13, 95% CI: 1.1–1.3, *p* = 0.04). Among the 50 patients with follow-up data, 68% achieved a term live birth, with no significant difference between the MTX (52.9%) and surgical (75.8%) groups (*p* 0.12). Most of the pregnancies (90%) occurred spontaneously, while only 10% required assisted reproductive technologies. *Conclusions*: MTX is a safe and effective treatment for tubal EP when patients are appropriately selected. Gestational sac diameter appears to be a reliable predictor of success. Both medical and surgical treatments yielded comparable reproductive outcomes, supporting individualized care models that prioritize fertility preservation.

## 1. Introduction

Ectopic pregnancy (EP) is a potentially life-threatening condition, affecting approximately 1–2% of all pregnancies and representing the leading cause of maternal mortality in the first trimester [1,2]. Despite diagnostic and therapeutic advances, EP continues to represent a major clinical challenge in early pregnancy care [3].

Timely diagnosis is crucial to avoid severe complications such as tubal rupture and hemoperitoneum. The clinical presentation may include pelvic pain, abnormal uterine bleeding, or signs of hemodynamic instability [4,5]. However, symptoms are often subtle and nonspecific, which may lead to delayed recognition [6,7]. The majority of ectopic pregnancies, approximately 95%, occur in the fallopian tube [8]. Known risk factors include pelvic inflammatory disease, previous pelvic surgery, smoking, endometriosis, use of intrauterine devices, infertility, and prior ectopic pregnancy [8,9,10].

Diagnosis is typically established through transvaginal ultrasound with serial serum β-hCG measurements [5,11]. Although a definitive diagnosis is confirmed by visualizing a gestational sac outside the uterine cavity, most cases are identified earlier based on clinical and biochemical findings [12].

Methotrexate (MTX), a folate antagonist, was introduced for the medical management of ectopic pregnancy in the late 1980s. Early studies demonstrated its efficacy as a non-surgical option in hemodynamically stable women [13]. Systemic MTX administration is now widely used in this setting, although specific eligibility criteria vary across guidelines. Generally, gestational sac dimensions, embryo visualization, the presence or absence of cardiac activity, free fluid in the pelvis, and β-hCG levels are considered key factors for treatment decisions and prognosis, but cut-offs differ. The American College of Obstetricians and Gynecologists (ACOG) and the Collège National des Gynécologues et Obstétriciens Français (CNGOF) consider treatment appropriate up to β-hCG levels of 5000 mIU/mL and with adnexal masses ≤ 3.5–4 cm [11,14]. In contrast, the National Institute for Health and Care Excellence (NICE) and the Royal College of Obstetricians and Gynaecologists (RCOG) adopt a more conservative threshold, recommending Methotrexate primarily when the β-hCG is < 1500 mIU/mL [5,15]. In clinical practice, local protocols may further modulate these thresholds based on institutional policies, resource availability, and clinical experience. While fertility preservation is a critical concern, and data support the safety of MTX, surgery may still be preferred in some clinical scenarios due to institutional preferences, experience, clinician judgment, or anatomical considerations [16,17].

Surgical management is indicated for patients who are not eligible for, or decline, medical treatment. It includes both salpingostomy and salpingectomy, most commonly performed via laparoscopy, and is the recommended choice in hemodynamically unstable cases [5,11,14].

A history of previous ectopic pregnancy is one of the strongest predictors of recurrence, with rates up to 25% in women with multiple prior events [18]. This risk is particularly relevant in young women with reproductive desire, for whom treatment choice may impact future fertility [9]. The preservation of reproductive potential has become a priority in the management of reproductive-age patients, with growing interest in fertility-sparing approaches even outside oncological settings [19,20,21]. Therefore, selecting the appropriate therapeutic approach requires balancing efficacy, safety, and reproductive outcomes.

Despite the availability of well-established treatment protocols, the management of tubal ectopic pregnancy remains highly variable across clinical settings [22]. Differences in guideline recommendations, institutional policies, and clinical experience contribute to heterogeneous therapeutic approaches. This variability is particularly evident in the choice between medical and surgical management.

The primary aim of this study is to evaluate the efficacy of conservative management of tubal ectopic pregnancy in comparison with surgical treatment. We also aimed to assess the proportion of patients requiring surgical intervention following the failure of medical therapy. A secondary objective is to assess fertility outcomes in women with reproductive desire by comparing the rates of live birth pregnancies between patients treated medically and those treated surgically. Furthermore, the study investigates whether these pregnancies were achieved spontaneously or through assisted reproductive techniques.

## 2. Materials and Methods

This retrospective, single-center study was conducted at the Obstetrics and Gynecology Unit of the University Hospital of Parma. Women aged 18 years or older with a diagnosis of tubal ectopic pregnancy, treated either medically or surgically between January 2019 and December 2023, were included. Data were collected from medical records between March and July 2024.

Collected variables included patient age, body mass index (BMI), parity, and risk factors for ectopic pregnancy such as prior pelvic surgery, previous ectopic pregnancy, pelvic inflammatory disease, and spontaneous miscarriage. Obstetric data included gestational age at diagnosis, β-hCG levels, and gestational sac diameter.

Patients were treated according to the hospital’s internal protocol. Medical management with intramuscular Methotrexate (50 mg/m^2^) was offered to hemodynamically stable patients presenting with serum β-hCG levels < 5000 mIU/mL and/or an adnexal mass < 40 mm, absence of embryonic cardiac activity, and no ultrasonographic evidence of hemoperitoneum. β-hCG levels were re-evaluated on days 4 and 7 post-treatment. A decrease of less than 20% between these time points was considered treatment failure, prompting either a second Methotrexate dose or surgical intervention, based on clinical judgment and patient preference. Surgical management, typically salpingectomy, was indicated in cases of β-hCG ≥ 5000 mIU/mL, adnexal mass > 40 mm, presence of embryonic cardiac activity, hemodynamic instability, or suspected tubal rupture.

Fertility outcomes were assessed by contacting patients by phone call, to determine whether a subsequent pregnancy occurred and whether it was achieved spontaneously or through assisted reproductive technology. Follow-up data regarding reproductive outcomes were obtained through structured telephone interviews with participants. Only pregnancies resulting in live births were collected. No imputation was performed for missing fertility outcomes.

The primary objective was to evaluate the efficacy of medical versus surgical management, including the rate of second Methotrexate administration or surgical intervention following medical treatment failure. Secondary objectives included the assessment of live birth rates and the mode of conception in women with reproductive desire.

Continuous variables were reported as mean and standard deviation (SD), and categorical variables as frequency and percentage. Normality of continuous variables was assessed using the Shapiro–Wilk test. Depending on distribution, continuous variables were compared using either the independent samples *t*-test or the Mann–Whitney U test. Categorical variables were compared using the chi-squared (χ^2^) test. Predictors of Methotrexate failure were analyzed using univariate and multivariate logistic regression models. Receiver operating characteristic (ROC) curve analysis was performed to identify clinically useful cut-off values. An area under the curve (AUC) of 0.7 or higher was considered acceptable for diagnostic performance. Statistical analyses were performed using IBM SPSS Statistics, version 28.0.1.1 (IBM Corp., Armonk, NY, USA).

The study protocol was approved by the local Ethics Committee, approval number 687/2023/OSS/AOUPR, and conducted in accordance with current regulations on patient privacy and data protection.

## 3. Results

### 3.1. Overall Cohort

Between 2019 and 2023, a total of 107 patients with a diagnosis of tubal ectopic pregnancy were treated at the Obstetrics and Gynecology Unit of the University Hospital of Parma.

The mean age of the cohort was 32.7 years (SD ± 5.7), with an average body mass index (BMI) of 23.5 kg/m^2^ (SD ± 5.3). In terms of parity, 47.7% of patients were nulliparous, while 52.3% had experienced at least one previous pregnancy. A prior ectopic pregnancy was reported in 14.9% of cases, spontaneous miscarriage in 17.7%, and recurrent pregnancy loss in 5.6%. Previous pelvic surgery, including procedures involving the uterus, adnexa, or cesarean sections, was reported in 36.4% of patients (Table 1).

At diagnosis, the mean gestational age was 41.3 days (SD ± 13.3 days), the mean initial serum β-hCG level was 8441.8 mIU/mL, and the average gestational sac diameter was 22.37 mm (Table 2). The majority of the patients (84/107; 78%) were symptomatic at presentation: in total, 14 (13.0%) reported vaginal bleeding, 62 (58.0%) had pelvic pain, and 8 (7.5%) showed signs of hemodynamic instability.

### 3.2. Medical and Surgical Treatment

Of the total cohort, 71 patients (66.4%) underwent surgical management via salpingectomy, while 36 (33.6%) received medical treatment with intramuscular MTX.

Among the patients treated with MTX (n = 36), 26 (72.2%) achieved resolution of the ectopic pregnancy: a total of 20 responded to a single dose, while 6 required a second dose. Surgical intervention was necessary in 10 patients (27.8%) due to treatment failure (Table 3).

The baseline characteristics such as age, BMI, parity, and obstetric history were comparable between the MTX and surgical groups (Table 4).

However, significant differences were observed in clinical presentation at diagnosis. The patients undergoing surgery had significantly higher initial β-hCG levels (12,216.8 vs. 2189 mIU/mL, *p* = 0.002), larger gestational sac diameters (27.2 mm vs. 14.8 mm, *p* = 0.010), and were more frequently symptomatic (86% vs. 61%, *p* = 0.005), particularly with pelvic pain (67.6% vs. 38.9%) and signs of hemodynamic compromise (9.9% vs. 0%) (Table 5).

### 3.3. Methotrexate Subgroup Analysis

A subgroup analysis compared the patients who successfully responded to MTX (n = 26) with those who required surgical intervention following treatment failure (n = 10). No significant differences were found in age, BMI, initial β-hCG levels, or prior obstetric or surgical history (Table 6).

The patients who failed MTX therapy had significantly larger gestational sac diameters (33.2 mm vs. 16.4 mm, *p* = 0.04). A logistic regression analysis identified sac diameter as the only factor significantly associated with MTX failure (OR 1.13, 95% CI 1.1–1.3, *p* = 0.04), a finding confirmed in multivariate analysis (OR 1.13, 95% CI 1.01–1.3, *p* = 0.04) (Table 7 and Table 8).

**Figure 1:**
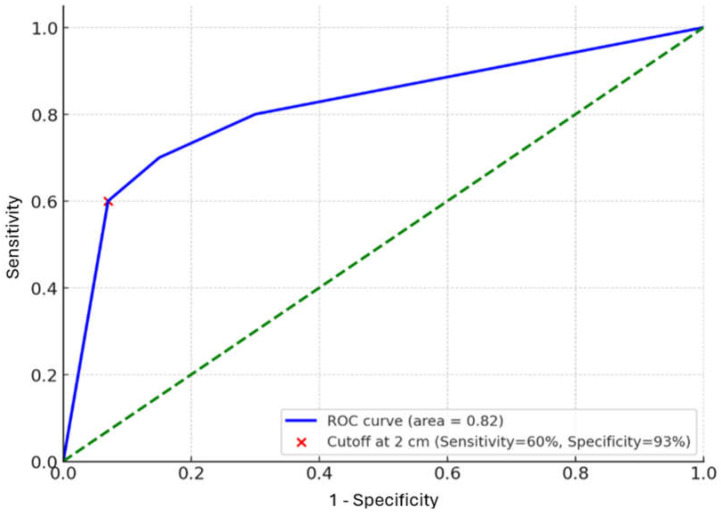
ROC Curve for Gestational Sac Diameter and Methotrexate Failure (n = 36). Abbreviations: ROC = Receiver Operating Characteristic; MTX = Methotrexate.

A receiver operating characteristic (ROC) curve analysis identified a gestational sac diameter > 20 mm as predictive of MTX failure, with 60% sensitivity, 93% specificity, and an area under the curve (AUC) of 0.82 (Figure 1).

### 3.4. Fertility Outcomes

Follow-up data on fertility outcomes were obtained for 50 patients (46.7% of the cohort). Of these, 17 had been treated with MTX and 33 with salpingectomy. Overall, 68% (34/50) achieved a subsequent live birth.

In the MTX group, 9 of 17 patients (52.9%) had a live birth, all of which occurred spontaneously. In the surgical group, 25 of 33 patients (75.8%) achieved a live birth, with 80% of those pregnancies conceived spontaneously and 20% via assisted reproductive technologies (Table 9). The time to conception was similar in both groups (16 vs. 18.6 months).

Although a higher live birth rate was observed in the surgical group, the difference was not statistically significant (*p* 0.12). Both approaches yielded comparable reproductive outcomes in terms of pregnancy and live birth.

### 3.5. Treatment Trends over Time

**Figure 2:**
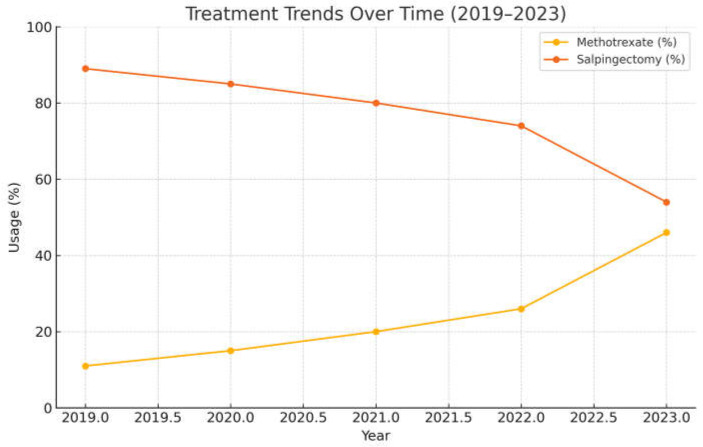
Annual Trends in Methotrexate and Surgical Treatment (2019–2023).

A temporal analysis from 2019 to 2023 showed a progressive shift toward conservative management. MTX usage increased from 11% in 2019 to 46% in 2023, while surgical treatment decreased from 89.3% to 54% (Figure 2).

The most notable increase in MTX use occurred between 2022 and 2023, with a 20% rise. This trend likely reflects growing clinical experience and confidence with medical protocols for ectopic pregnancy management in our center.

## 4. Discussion

### 4.1. Summary of Main Results

This retrospective study evaluated the clinical characteristics and treatment outcomes of 107 patients with tubal ectopic pregnancy managed at the University Hospital of Parma.

The MTX success rate was 72%, aligning with published data. The patients treated surgically presented with significantly higher β-hCG levels, larger gestational sacs, and more frequent symptoms, including pelvic pain and hemodynamic instability, consistent with institutional criteria. A sixfold difference in initial β-hCG levels was observed between the patients treated medically and those undergoing primary surgery, reflecting the pre-established selection criteria that stratify patients into two clinically distinct groups (see Table 5).

The demographic and obstetric variables were comparable between the treatment groups. A previous ectopic pregnancy did not influence treatment allocation or outcome.

Gestational sac diameter was the strongest predictor of MTX failure. The ROC curve analysis identified a threshold of >20 mm (AUC 0.82, sensitivity 60%, specificity 93%).

Among the patients receiving MTX, 20 responded to a single dose, and 6 required retreatment. No significant associations were found between MTX failure and age, BMI, β-hCG level, or gestational age. Notably, some successful cases involved β-hCG levels > 5000 mIU/mL, suggesting that MTX may remain a viable option in selected cases beyond conventional thresholds.

Approximately one-third of the patients required retreatment. No demographic or clinical variables were associated with this need. Conditions such as endometriosis, fibroids, or prior pelvic surgery were not statistically significant but may warrant further study.

### 4.2. Results in the Context of the Published Literature

Despite the availability of evidence on the efficacy of both medical and surgical approaches in the management of tubal ectopic pregnancy, the selection criteria for MTX remain inconsistent across international guidelines, particularly regarding β-hCG thresholds and gestational sac dimensions [5,11,14]. While β-hCG levels below 1500 mIU/mL are considered optimal for medical management due to their association with lower retreatment rates [23], robust evidence supports the safety of repeated MTX dosing. This has allowed treatment to be safely extended to patients with levels up to 5000 mIU/mL who meet appropriate clinical criteria [7].

Our study contributes to this discussion by reporting real-world outcomes from a referral center applying standardized protocols. We also explored clinical and sonographic predictors of medical treatment success that may support the refinement of current eligibility criteria.

An important aspect emerging from our analysis concerns the evaluation of predictive factors for MTX success. Although the existing literature suggests that elevated β-hCG levels (>5000 mIU/mL) are associated with increased risk of treatment failure [23,24], and current guidelines discourage MTX use in this setting [5,11,14], our results support the need for an integrated evaluation of treatment eligibility. In our cohort, the patients who responded successfully to MTX had, on average, higher initial β-hCG levels compared to those who experienced treatment failure. However, this difference was not statistically significant and should be interpreted as a descriptive finding rather than a clinically relevant association. Given the substantial overlap in β-hCG values between responders and non-responders, mean levels alone are not appropriate as decision-making thresholds. Nevertheless, these findings suggest that while higher β-hCG levels may reduce MTX efficacy, they do not entirely preclude successful medical management [24]. Consequently, rigid exclusion criteria based solely on β-hCG may unnecessarily restrict patient eligibility for conservative treatment. Notably, our data indicated that, in this cohort, only gestational sac diameter appeared to predict failure of a single MTX administration. However, we acknowledge that the small sample size limits the power of this finding, and the role of β-hCG cannot be definitively excluded.

We hypothesize that due to the wide range of β-hCG values and the variability in metabolic activity of trophoblastic tissue, β-hCG alone may not accurately reflect the biological behavior or maturity of the ectopic pregnancy. In contrast, gestational sac diameter might more reliably indicate the developmental stage and sensitivity to MTX therapy. The diameter of the gestational sac likely reflects the trophoblastic mass more accurately than β-hCG levels and may better predict the likelihood of successful resolution with systemic Methotrexate, particularly in the absence of an embryonic pole. Recently, a nomogram for the prediction of MTX success was proposed by Zeevi et al. based on a retrospective series of more than 300 patients treated with MTX for EP [25]. In this cohort, the presence of a fetal pole was associated with an odds ratio (OR) for MTX failure of 4.25 (95% CI: 1.05–17.2). The only other factor associated with MTX failure in the multivariable analysis was β-hCG level on day 1 after treatment administration; however, the clinical utility of this parameter is limited by its post-exposure nature. Interestingly, the impact of gestational sac diameter was not demonstrated [25] However, the mean gestational sac diameters in the population of the previous study were significantly smaller than in ours—14 mm and 13 mm in the success and failure groups, respectively. This may suggest that, in the clinical setting in [25], patients with smaller gestational sacs are more often selected for MTX treatment, possibly reflecting different selection criteria compared to our institution [25]. Nevertheless, supporting our hypothesis, the presence of a fetal pole may be a marker of a more metabolically active and viable ectopic pregnancy, possibly indicating reduced responsiveness to MTX more accurately than β-hCG levels alone. In fact, it was previously reported that, even in intrauterine pregnancies, gestational sac dimensions correlate more strongly with viability than single-point β-hCG measurements, as both endogenous and analytical factors can influence β-hCG levels [26,27].

Given the increasing attention to fertility preservation, particularly in young women of reproductive age affected by both non-oncologic and oncologic conditions [28,29], the comparative effectiveness of medical versus surgical management has been widely investigated, especially in terms of reproductive outcomes. The DEMETER trial, a randomized controlled study involving 400 patients conducted in France, found no significant differences in two-year spontaneous pregnancy rates between patients treated with MTX and those managed surgically, with live birth rates of 67% and 64–70%, respectively. Moreover, no advantage was found in salpingostomy vs. salpingectomy [30]. Similarly, the European Surgery in Ectopic Pregnancy (ESEP) multicenter trial compared salpingectomy and salpingostomy in women with a healthy contralateral tube, showing comparable cumulative pregnancy rates of 56.2% and 60.7%, respectively, and concluded no clear advantage of one surgical approach over the other [31]. In addition to limited evidence supporting its benefits, salpingostomy also has a non-negligible risk of persistent trophoblastic tissue (TRAP), which occurs in approximately 10% of cases [32]. In light of these findings, in our center, salpingectomy is the preferred surgical approach and was the treatment of choice in patients elected for surgery in our cohort.

Recent data from Düz et al. further confirmed the comparability of reproductive outcomes across treatment modalities [33]. Their 2022 study reported no significant differences in intrauterine pregnancy rates between patients treated with MTX, those undergoing primary surgery, and those requiring surgery following medical treatment failure. These findings support the notion that, in appropriately selected patients, conservative management is not inferior to surgical treatment in terms of fertility preservation.

Our findings are consistent with this study. Among the 50 patients in our cohort with available follow-up data, 68% achieved a term live birth, with no statistically significant difference between the MTX (52.9%) and surgical (75.8%) groups. Most of the pregnancies occurred spontaneously, and only 10% required assisted reproductive techniques. The numerically higher rate in the surgical group was not statistically significant, in line with the non-inferiority of MTX compared to radical surgery reported in the existing literature.

Over the course of the study period, we observed a progressive increase in the use of MTX, particularly after 2019. In 2019, only 11% of patients were treated medically, whereas this proportion rose to 46% by 2023. This trend suggests growing clinical confidence and institutional familiarity with conservative protocols. Furthermore, half of the surgical conversions following MTX failure occurred in 2019, while only one or two such cases were recorded annually in the subsequent years. These data reflect increased experience and protocol adherence, but also the implementation of international guidelines and growing evidence supporting the efficacy of repeated MTX administration, contributing to more accurate patient selection and improved treatment outcomes [34,35].

Taken together, these findings reinforce the growing consensus that both MTX and salpingectomy are valid and effective treatment options for tubal ectopic pregnancy, offering comparable fertility outcomes when applied according to clear clinical criteria. They also highlight the importance of individualized care, supported by accurate ultrasound assessment and appropriate counseling, in optimizing both clinical results and patient-centered goals.

### 4.3. Strengths and Limitations

The main strengths of this study include the homogeneity of clinical management, ensured by shared protocols within a single center, and the consistent application of eligibility criteria. The flexibility in β-hCG thresholds reflects real-life clinical reasoning and enhances the generalizability of the findings.

Another strength is the longitudinal observation of practice trends over five years, documenting the progressive adoption of conservative management and improved selection of candidates for MTX.

Finally, follow-up data on fertility outcomes, available for nearly half of the cohort, provide additional value by linking treatment strategies with long-term reproductive results. The follow-up data on fertility outcomes were collected through structured telephone interviews and included only pregnancies resulting in live births, a clinically meaningful endpoint in reproductive medicine.

Nonetheless, several limitations must be acknowledged. As a retrospective study, it is subject to potential data omissions and selection bias, including possible recall bias from follow-up interviews. The observed imbalance in the treatment groups may reflect a greater inclusion of patients not eligible for MTX during the study period, as well as the centralization of complex and emergency cases at our institution from nearby hospitals. Moreover, the relatively small sample size limits the statistical power, particularly for subgroup comparisons. Future prospective studies with larger cohorts would help validate our findings and refine treatment algorithms.

### 4.4. Implications for Future Research and Clinical Practice

Our findings suggest that sonographic parameters, particularly gestational sac diameter, are the strongest predictors of successful medical treatment. A diameter < 2 cm appears to be a strong predictor of MTX success, potentially improving the accuracy of pre-treatment counseling regarding the likelihood of resolution with medical therapy, and guiding subsequent decisions in the event of single-dose failure. Conversely, β-hCG levels above 5000 mIU/mL may unjustifiably discourage MTX use, despite favorable clinical conditions in selected patients. These findings provide a rationale for future prospective studies and may prompt a reconsideration of current guideline-endorsed criteria for choosing between MTX and salpingectomy. If validated, a broader eligibility framework for MTX could be proposed, wherein elevated β-hCG levels alone would not preclude conservative management in the presence of a permissive gestational sac diameter.

Given its accessibility and non-invasive nature, transvaginal ultrasound plays a key role in the initial evaluation and therapeutic planning of ectopic pregnancy [36]. Its routine use can enhance patient selection for conservative treatment and improve individualized care.

Future studies should aim to validate this cut-off in larger, prospective cohorts, and to explore additional clinical or biochemical predictors of MTX response. Long-term outcomes, including both reproductive and psychological well-being, should also be assessed. Incorporating these elements into patient counseling may support more individualized, fertility-preserving treatment strategies, aligned with patient values and reproductive goals.

## 5. Conclusions

Ectopic pregnancy remains a serious condition requiring timely diagnosis and appropriate intervention to prevent maternal morbidity and preserve reproductive potential. Both MTX and salpingectomy are effective options when patients are selected according to well-defined clinical criteria.

Our findings support the safety and efficacy of MTX in appropriately selected patients and emphasize the importance of transvaginal ultrasound evaluation and pre-treatment counseling in guiding therapeutic decisions. A gestational sac diameter < 20 mm appears to be a strong predictor of medical treatment success and may inform future protocol refinement.

Although based on a single-center, retrospective study, these results suggest that a small gestational sac diameter may help identify suitable candidates for MTX, even in the presence of elevated β-hCG levels, which alone should not necessarily preclude conservative treatment. Prospective, multicenter studies are warranted to validate these findings and support potential updates to current clinical guidelines.

Finally, this study supports the broader implementation of conservative management where feasible, and advocates for individualized care models that prioritize fertility preservation and patient-centered outcomes.

## Figures and Tables

**Table 1:** Demographic and Anamnestic Characteristics of the Patients.

Variable	Value (*n* 107)
Age (years)	32.7 ± 5.7
Weight (kg)	63.5 ± 14.7
BMI (kg/m^2^)	23.5 ± 5.3
Nulliparous (%)	51 (47.7%)
Previous miscarriage (%)	19 (17.7%)
Recurrent pregnancy loss (%)	6 (5.6%)
Previous ectopic pregnancy (%)	16 (14.9%)
Previous pelvic surgery (%)	39 (36.4%)

**Table 2:** Pregnancy Characteristics and Clinical Presentation.

Variable	Value
Gestational age (days)	41.3 ± 13.3
Initial β-hCG (mIU/mL)	8441.8 ± 20,582
Gestational sac diameter (mm)	22.4 ± 14.6
Symptomatic at diagnosis (%)	83 (78%)
Vaginal bleeding (%)	14 (13%)
Pelvic pain (%)	62 (58%)
Hemodynamic instability (%)	7 (7.5%)

**Table 3:** Outcomes of Methotrexate Treatment in Tubal Ectopic Pregnancy.

Treatment Modality	n (%) of Group	n (%) of Total; N = 107
Medical treatment	36	36 (33.6%)
Resolution after Methotrexate	26 (72%)	-
After 1 dose	20 (55.5%)	-
After 2 doses	6 (5.5%)	-
Surgical conversion after failure	10 (28%)	-
Surgical treatment	-	71 (66.35%)
Salpingectomy	-	71 (66.35%)

**Table 4:** Comparison of Anamnestic Characteristics in Patients Treated with Medical vs. Surgical Management.

Variable	Methotrexate (n = 36)	Salpingectomy (n = 71)	*p*-Value
Age (years)	32.3 ± 2.1	32.8 ± 6.1	0.09
Weight (kg)	63.8 ± 19.3	63.4 ± 12.8	0.68
BMI (kg/m^2^)	24.9 ± 4.5	23 ± 5.5	0.23
Nulliparous	16 (44.4%)	35 (49.3%)	0.69
Previous miscarriage	8 (22.2%)	11 (15.5%)	0.43
Recurrent pregnancy loss	2 (5.6%)	5 (5.6%)	1.00
Previous ectopic pregnancy	6 (16.7%)	10 (14.1%)	0.78
Previous pelvic surgery	15 (41.7%)	24 (33.8%)	0.52

**Table 5:** Clinical Characteristics of Patients Treated with Methotrexate vs. Surgical Salpingectomy.

Variable	Methotrexate	Salpingectomy	*p*-Value
Gestational age (days)	43.4 ± 9.1	46.9 ± 8.4	0.43 *
Initial β-hCG (mIU/mL)	2189 ± 2244	12,216.8 ± 25,776	<0.001 *
Gestational sac diameter (mm)	14.8 ± 3.1	27.2 ± 15.4	0.02 *
Symptomatic at diagnosis (%)	22 (61%)	61 (86%)	0.005
Vaginal bleeding (%)	8 (22.2%)	6 (8.5%)	0.001
Pelvic pain (%)	14 (38.9%)	48 (67.6%)	0.001
Hemodynamic instability (%)	0	7 (9.9%)	0.001

* *p*-values calculated using the Mann–Whitney U test.

**Table 6:** Comparative Analysis of Parameters in Methotrexate-Treated Patients: Success vs. Surgical Conversion.

Variable	Resolved (n 26)	Surgery (n 10)	*p*-Value
Age (years)	32.6 ± 5.4	31.7 ± 4.21	0.49
Weight (kg)	61.6 ± 22.6	67 ± 13.9	0.25
BMI (kg/m^2^)	24.6 ± 3.9	25.3 ± 5.5	0.62
Gestational age (days)	43.4 ± 9.1	43.4 ± 8.4	0.73
β-hCG (mIU/mL)	2443 ± 2534	1527.9 ± 1038	0.14
Sac diameter (mm)	16.4 ± 6.1	33.2 ± 18.4	0.01 *
Prior pelvic surgery (%)	11 (42%)	4 (40%)	1.000
Prior ectopic pregnancy (%)	4 (15.4%)	2 (20%)	1.000
Symptomatic (%)	15 (57.7%)	7 (70%)	0.71

* *p*-values calculated using the Mann–Whitney U test.

**Table 7:** Univariate Logistic Regression Analysis: Predictors of Surgical Conversion After Methotrexate.

Variable	OR	95% CI	*p*-Value
BMI	1.2	0.9–1.4	0.13
Patient age	1.03	0.9–1.2	0.6
Gestational age	1.6	0.9–2.6	0.09
Weight	1.04	0.9–1.1	0.2
Sac diameter	1.13	1.1–1.3	0.04
β-hCG at diagnosis	1	-	0.3
Pelvic surgery	2.79	0.47–16.34	0.42
Symptoms at diagnosis	2.1	0.39–11.43	0.39

Abbreviations: BMI = Body Mass Index; MTX = Methotrexate; OR = Odds Ratio; CI = Confidence Interval.

**Table 8:** Multivariate Logistic Regression Analysis: Predictors of Surgical Conversion After Methotrexate.

Variable	OR	95% CI	*p*-Value
Gestational age	1.9	0.5–5.1	0.19
Sac diameter	1.13	1.01–1.3	0.04

Abbreviations: OR = Odds Ratio; CI = Confidence Interval.

**Table 9:** Fertility Outcomes in Patients Treated with Methotrexate vs. Salpingectomy.

Outcome	Methotrexate	Salpingectomy	*p*-Value
Live birth rate	9/17 (52.9%)	25/33 (75.8%)	0.12
Spontaneous conception	9 (100%)	20/25 (80%)	0.29
Time to conception (months)	16 ± 8.5	18.6 ± 9.9	0.09

## Data Availability

The original contributions presented in this study are included in the article. Further inquiries can be directed to the corresponding author.
